# The prognostic values of EGFR expression and *KRAS* mutation in patients with synchronous or metachronous metastatic colorectal cancer

**DOI:** 10.1186/1471-2407-13-599

**Published:** 2013-12-13

**Authors:** Ching-Wen Huang, Hsiang-Lin Tsai, Yi-Ting Chen, Chun-Ming Huang, Cheng-Jen Ma, Chien-Yu Lu, Chao-Hung Kuo, Deng-Chyang Wu, Chee-Yin Chai, Jaw-Yuan Wang

**Affiliations:** 1 Graduate Institute of Medicine, College of Medicine, Kaohsiung Medical University, Kaohsiung, Taiwan; 2 Department of Surgery, Kaohsiung Municipal Hsiao-Kang Hospital, Kaohsiung Medical University, Kaohsiung, Taiwan; 3 Division of Gastrointestinal and General Surgery, Department of Surgery, Kaohsiung Medical University Hospital, Kaohsiung Medical University, Kaohsiung 807, Taiwan; 4 Cancer Center, Kaohsiung Medical University Hospital, Kaohsiung Medical University, Kaohsiung, Taiwan; 5 Division of General Surgery Medicine, Department of Surgery, Kaohsiung Medical University Hospital, Kaohsiung, Taiwan; 6 Program of Bachelor of Health Beauty, School of Medical and Health Sciences, Fooyin University, Kaohsiung, Taiwan; 7 Department of Pathology, Kaohsiung Medical University Hospital, Kaohsiung Medical University, Kaohsiung, Taiwan; 8 Department of Radiation Oncology, Kaohsiung Medical University Hospital, Kaohsiung, Taiwan; 9 Graduate Institute of Clinical Medicine, College of Medicine, Kaohsiung Medical University, Kaohsiung, Taiwan; 10 Division of Gastroenterology, Department of Internal Medicine, Kaohsiung Medical University Hospital, Kaohsiung Medical University, Kaohsiung, Taiwan; 11 Department of Internal Medicine, Faculty of Medicine, College of Medicine, Kaohsiung Medical University, Kaohsiung, Taiwan; 12 Department of Pathology, Faculty of Medicine, College of Medicine, Kaohsiung Medical University, Kaohsiung, Taiwan; 13 Department of Surgery, Faculty of Medicine, College of Medicine, Kaohsiung Medical University, Kaohsiung, Taiwan

**Keywords:** Epidermal growth factor receptor, *KRAS*, Prognostic value, Metachronous, Synchronous, Metastatic colorectal cancer

## Abstract

**Background:**

The epidermal growth factor receptor (EGFR)/RAS/RAF/MEK/MAPK pathway is an important pathway in the carcinogenesis, invasion and metastasis of colorectal cancers (CRCs). We conducted a retrospective study to determine the prognostic values of EGFR expression and *KRAS* mutation in patients with metastatic CRC (mCRC) based on synchronous or metachronous status.

**Methods:**

From October 2002 to March 2012, 205 patients with mCRC were retrospectively analyzed; 98 were found to have metachronous mCRC while 107 were found to have synchronous mCRC. The EGFR expressions were determinate by IHC (immunohistochemistry) analysis and categorized 1+ (weak intensity), 2+ (moderate intensity), and 3+ (strong intensity). Genomic DNA was isolated from frozen primary CRC tissues and direct sequencing of *KRAS* was performed. The clinicopathological features of these mCRC patients were retrospectively investigated according to EGFR expression and *KRAS* mutation status. Moreover, we analyzed the prognostic values of EGFR expression and *KRAS* mutation among these patients.

**Results:**

Of the 205 patients with mCRC, EGFR expression was analyzed in 167 patients, and positive EGFR expression was noted in 140 of those patients (83.8%). *KRAS* mutation was investigated in 205 patients and mutations were noted in 88 of those patients (42.9%). In patients with metachronous mCRC, positive EGFR expression was significantly correlated with well-and moderately-differentiated tumors (*P* = 0.028), poorer disease-free survival (DFS) (*P* < 0.001), and overall survival (OS) (*P* < 0.001). Furthermore, positive EGFR expression was a significant independent prognostic factor of DFS (*P* = 0.006, HR: 4.012, 95% CI: 1.130–8.445) and OS (*P* = 0.028, HR: 3.090, 95% CI: 1.477–10.900) in metachronous mCRC patients. *KRAS* mutation status was not significantly related to DFS and OS of patients with metachronous mCRC; likewise, *KRAS* mutation status was not significantly different in the progression-free survival (PFS) and OS of patients with synchronous mCRC (all *P* > 0.05).

**Conclusions:**

The present study demonstrated that EGFR expression has prognostic value only for patients with metachronous mCRC. However, *KRAS* mutation did not have prognostic value in patients with metachronous or synchronous mCRC.

## Background

Colorectal cancer (CRC) is the third most common cancer and the third leading cause of cancer death in the United States where an estimated 142,820 newly diagnosed cases of CRC and an estimated 50,830 cancer deaths from CRC were reported in 2013 [1]. In Taiwan, CRC is the most common cancer type, having increased rapidly in prevalence, and the third leading cause of cancer-related death as of 2012. The incidence of CRC was 32.38 per 100,000 (7,213 new diagnoses of CRC) in 2000 and 60.72 per 100,000 (14,040 new diagnoses of CRC) in 2010 [2]. In Taiwan, 5131 people died from CRC in 2012 and the death rate was 22.0 per 100,000 [2]. The prognoses of metastasis colorectal cancer (mCRC) have improved in the past decade, with the median overall survival (OS) rate increasing from 12 months to more than 24 months [3,4]. These improvements are considered to be a result of the development of combinations of standard chemotherapy, including fluoropyrimidine/folinic acid, irinotecan (FOLFIRI), and oxaliplatin (FOLFOX), and the introduction of new targeted biological agents such as cetuximab, panitumumab, and bevacizumab.

EGFR is a 170-KDa transmembrane receptor with an intracellular tyrosine kinase domain. EGFR is a member of the ErbB receptor family. After EFGR is bounded by EGF, EFGR forms a functionally active dimer (homodimer or heterodimer) that causes phosphorylation of tyrosine kinases in the intracellular domain of EGFR. Subsequently, complex intracellular signals to the cytoplasm and then to the nucleus are triggered by this phosphorylation [5]. Two major downstream signaling pathways are mediated by EGFR: the RAS/RAF/MEK/MAPK pathway and the PI3K–Akt pathway. The functions of the EGFR/RAS/RAF/MEK/MAPK pathway are associated with gene transcription, cell-cycle progression from the G1 phase to the S phase, and cell proliferation. Moreover, the EGFR/RAS/RAF/MEK/MAPK pathway has also been reported to play a critical role in the carcinogenesis, migration, invasion, and metastasis of CRC [5]. EGFR overexpression was previously thought to be associated with more advanced disease and worse prognoses. The prognostic value of EGFR in CRC has been investigated extensively, but it remains controversial [6-10]. Although *KRAS* mutation has been studied for the predictive value of tumor response to anti-EGFR treatment and also has been confirmed to be the highly predictive of resistance to anti-EGFR treatment [11-18], the prognostic value of *KRAS* mutation in synchronous and metachronous mCRC remains controversial [18-28]. Therefore, we conducted a retrospective study to evaluate the prognostic value of EGFR expression and KRAS mutation in patients with synchronous or metachronous mCRC. Synchronous metastasis was defined as metastatic disease at the time of the primary CRC diagnosis. Metachronous metastasis was defined as the absence of metastatic disease at the time of initial CRC diagnosis with metastatic disease developing more than 3 months after resection of the primary CRC.

## Methods

### Patients

This retrospective study included 205 patients with histologically proven synchronous or metachronous mCRC who received surgical treatment from a single-institution between October 2002 and July 2012. The present study was approved by the Institutional Review Board of the Kaohsiung Medical University Hospital. Patients’ clinical outcomes and survival statuses were regularly followed up. Available variables included: age of diagnosis, sex, tumor location, histological type, TNM classification, vascular invasion, perineural invasion, and preoperative and postoperative serum level of CEA. The TNM classification was defined according to the criteria of the American Joint Commission on Cancer/International Union Against Cancer (AJCC/UICC) [29]. All patients were followed up until their deaths, their last follow-up, or December 31, 2012. Overall survival (OS) was defined as the time from the date of primary treatment to the date of death from any cause or until the date of the last follow-up. Disease-free survival (DFS) for patients with metachronous mCRC was defined as the time from the date of primary treatment to the date of diagnosis for recurrence or metastatic disease or to the date of the last follow-up. Progress-free survival (PFS) for patients with synchronous mCRC was defined as the time from the date of primary treatment to the date of tumor progression or to the date of death from any cause, or to the date of the last follow-up.

### Immunohistochemical analysis for EGFR expression

Formalin-fixed and paraffin-embedded tissue blocks were cut into 3 μm sections and deparaffinized, rehydrated, and autoclaved at 121°C for 5 min in Target Retrieval solution (Dako, Glostrup, Denmark), pH 6.0, to retrieve antigens. Endogenous peroxidase was blocked by 3% hydrogen peroxide for 5 min at room temperature. After washing with a Tris buffer solution, the sections were incubated with EGFR for 1 hour at room temperature. Then, DAKO REAL EnVision Detection System-HRP (DAKO, Glostrup, Denmark) was applied for 30 minutes at room temperature. Finally, sections were incubated in 3′, 3-diaminobenzidine for 5 minutes, followed by Mayer’s hematoxylin counterstaining. Dehydration was performed through two changes of 95% ethanol and two changes of 100% ethanol, and the samples were cleared in three changes of xylene and then mounted. Negative controls were obtained by replacing the primary antibody with non-immune serum. Immunoreactivity of EGFR was evaluated by two independent researchers who were blinded to patient outcome.

**Figure 1:**
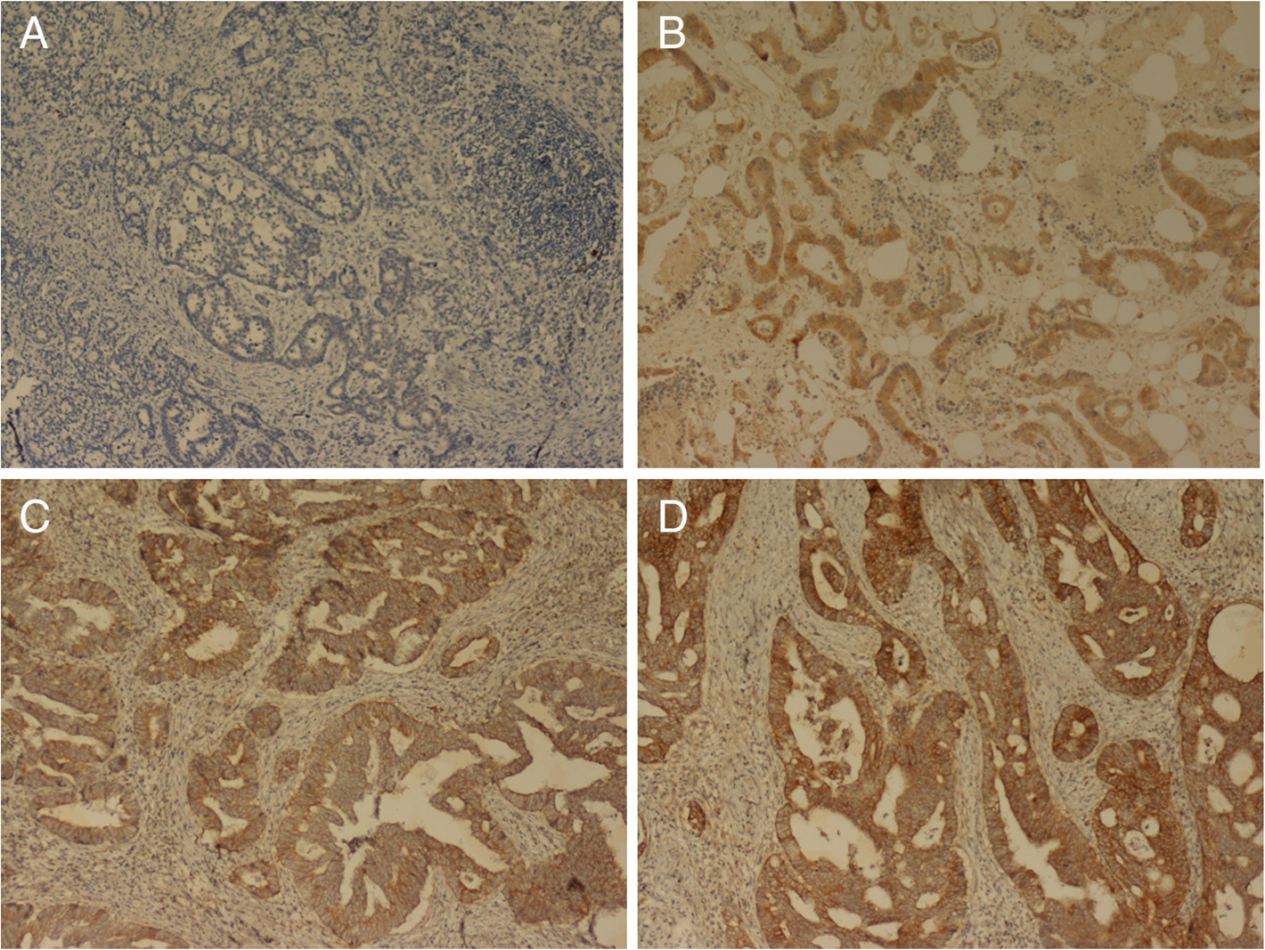
**Immunohistochemical staining of EGFR in CRC. A**. negative expression (magnification, 100X). **B**. 1+ (weak intensity of membrane staining) (magnification, 100X). **C**. 2+ (moderate intensity of membrane staining) (magnification, 100X). **D**. 3+ (strong intensity of membrane staining) (magnification, 100X).

Expression patterns of EGFR were determined in a semi-quantitative manner by light microscopy. Immunoreactivity for EGFR (membrane staining) was categorized in accordance with the presence of tumor cell staining and staining intensity. The intensity of EGFR immunoreactivity was scored with a 3-tier system as follow [7,30]: 1+ (weak intensity); 2+ (moderate intensity); and 3+ (strong intensity) (Figure 1). Negative EGFR expression means absence of membrane staining above background in all tumor cells. Positive EGFR expression is defined as any IHC (immunohistochemistry) complete or incomplete membrane staining of tumor cells, including intensity 1+, 2+ or 3 +.

### DNA extraction and direct sequencing of *KRAS*

Genomic DNA was isolated from frozen primary CRC tissues, using proteinase-K (Stratagene, La Jolla, CA, USA) digestion and the phenol/chloroform extraction procedure according to the method outlined by Sambrook et al. [31]. The designed sequences of oligonucleotide primers for exons 2 and 3 of the *KRAS* and the operational procedure of direct sequencing were based on those of our previously study [18,32].

### Statistical analysis

All data were statistically analyzed using the Statistical Package for the Social Sciences, version 19.0 (SPSS Inc., Chicago, IL, USA). The correlation between clinicopathological features and EGFR expression or *KRAS* mutation was compared using a Chi-square test (for categorical variables) and Student t-test (for continuous variables). The Cox proportional-hazards model was used for univariate and multivariate analyses to identify the independent prognostic factors for OS, DFS and PFS. OS, DFS, and PFS were calculated by the Kaplan-Meier method, and the differences in survival rates were analyzed by the log-rank test. A *P* value less than 0.05 was considered to be statistically significant.

## Results

### Characteristics of patients with mCRC

Of the 205 patients with mCRC, 98 patients (47.8%) were metachronous and 107 patients (52.2%) were synchronous. The mean age of the 205 patients was 61.0 ± 12.8 (range, 29–86) years of age. There were 120 males and 85 females. The median follow-up time for the 205 patients was 30.2 ± 20.9 (range, 1–137.3) months. Immunohistochemical analyses for EGFR expression were performed in 174 patients and positive expression was noted in 140 of those patients (80.5%). *KRAS* mutation status was evaluated in 205 patients, and mutation was noted in 88 of those patients (42.9%).

### Characteristics of patients with metachronous mCRC

The clinical and pathological data regarding the 98 patients with metachronous mCRC are summarized in Table 1. Immunohistochemical analyses for EGFR expression were performed in 84 patients, and positive EGFR expression was noted in 67 of those patients (79.8%). There were no significant differences in mean ages, gender, tumor location, AJCC/UICC cancer stage, retrieved lymph node number, vascular invasion, perineural invasion, pre-operative serum CEA level, and post-operative serum CEA level between patients with positive EGFR expression and those with negative EGFR expression. However, OS (36.72 vs. 62.51 months, *P* < 0.001) and DFS (14.48 vs. 34.27 months, *P* < 0.001) rates of patients with positive EGFR expression were significantly poorer than those of patients with negative EGFR expression.

**Table 1:** **Baseline characteristics of metachronous metastatic colorectal cancer patients by EGFR expression and *KRAS *** **mutation status**

**Characteristic**	**EGFR positive (%)**	**EGFR negative (%)**	***P *** **value**	***KRAS *** **WT**^**a **^**(%)**	***KRAS *** **Mut**^**b **^**(%)**	***P *** **value**
	**N = 67 (79.8%)**	**N = 17 (20.2%)**		**N = 54 (55.1%)**	**N = 44 (44.9%)**	
**Age (years, mean ± SD)**	59.13 ± 10.49	64.41 ± 12.13	0.076	59.98 ± 10.21	59.18 ± 13.81	0.743
**Gender**			0.547			0.011
Male	37 (55.2)	8 (47.1)		36 (66.7)	18 (40.9)	
Female	30 (44.8)	9 (52.9)		18 (33.3)	26 (59.1)	
**Tumor size**			0.04			0.910
≥5 cm	18 (26.9)	9 (53.9)		19 (35.2)	15 (34.1)	
<5 cm	49 (73.1)	8 (47.1)		35 (64.8)	29 (65.9)	
**Tumor location**			0.518			0.130
Colon	45 (67.2)	10 (58.8)		30 (49.2)	31 (34.0)	
Rectum	22 (32.8)	7 (41.2)		24 (64.9)	13 (66.0)	
**Histology**			0.044			0.688
Well	1 (2.5)	1 (5.9)		1 (1.9)	1 (2.3)	
Moderately	62 (92.5)	12 (70.6)		45 (83.3)	39 (88.6)	
Poorly	4 (5.0)	4 (23.5)		8 (14.8)	4 (9.1)	
**Histology**			0.028			0.390
Well + Moderately	63 (94.0)	13 (76.5)		46 (85.2)	40 (90.9)	
Poorly	4 (6.0)	4 (23.5)		8 (14.8)	4 (9.1)	
**AJCC stage (Initial diagnosis)**			0.928			0.993
I	6 (9.0)	2 (11.8)		5 (9.3)	4 (9.1)	
II	19 (28.4)	5 (29.4)		14 (25.9)	11 (25.0)	
III	42 (62.6)	10 (58.8)		35 (64.8)	29 (65.9)	
**Tumor depth**			0.531			0.344
T1	0 (0)	0 (0)		1 (1.9)	0 (0.0)	
T2	8 (11.9)	2 (11.8)		4 (7.4)	6 (13.6)	
T3	48 (71.7)	14 (82.3)		39 (72.2)	34 (77.3)	
T4	11 (16.4)	1 (5.9)		10 (18.5)	4 (9.1)	
**Lymph nodes metastases**			0.755			0.401
N0	25 (37.3)	7 (41.2)		19 (35.2)	15 (34.1)	
N1	30 (44.8)	6 (35.3)		21 (38.9)	22 (50.0)	
N2	12 (17.9)	4 (23.5)		14 (25.9)	7 (15.9)	
**Retrived LN**^**c**^	14.50 ± 8.70	16.00 ± 7.18	0.594	14.98 ± 9.98	15.69 ± 9.26	0.742
**Vascular invasion**			0.701			0.053
Yes	23 (34.3)	5 (29.4)		25 (46.3)	12 (27.3)	
No	44 (65.75)	12 (70.6)		29 (53.7)	32 (72.7)	
**Perineurial invasion**			0.395			0.624
Yes	23 (34.3)	4 (23.5)		21 (38.9)	15 (34.1)	
No	44 (65.75)	13 (76.5)		33 (61.1)	29 (65.9)	
**Pre-op serum CEA level**			0.598			0.482
≥5 ng/ml	27 (40.3)	7 (41.2)		24 (44.4)	21 (47.7)	
<5 ng/ml	40 (59.7)	10 (58.8)		30 (55.6)	23 (52.3)	
**Post-op serum CEA level**			0.356			0.097
≥5 ng/ml	21 (31.3)	4 (23.5)		20 (37.0)	10 (22.7)	
<5 ng/ml	46 (68.7)	13 (76.5)		34 (63.0)	34 (77.3)	
**Overall survival (months)**	36.72 ± 18.68	62.51 ± 31.86	<0.001	42.54 ± 28.07	37.41 ± 17.23	0.293
**Disease-free survival (months)**	14.48 ± 9.91	34.27 ± 18.58	<0.001	18.38 ± 15.86	16.43 ± 10.58	0.487

^a^WT: wild type; ^b^Mut: mutation; ^c^LN: lymph node.

*KRAS* mutation status was evaluated in 98 patients and mutation was noted in 44 of those patients (44.9%). There were no significant differences in mean ages, tumor location, histological type, AJCC/UICC cancer stage, retrieved lymph node number, vascular invasion, perineural invasion, pre-operative serum CEA level, and post-operative serum CEA level between patients with wild-type *KRAS* and those with mutated *KRAS*. OS (42.54 vs. 37.41 months, *P* = 0.293) and DFS (18.38 vs. 16.43 months, *P* = 0.487) were not significantly different between patients with wild-type *KRAS* and those with mutated *KRAS*.

### Characteristics of patients with synchronous mCRC

The clinical and pathological data regarding the 107 patients with synchronous mCRC are summarized in Table 2. Immunohistochemical analyses for EGFR expression were performed in 90 patients, and a positive EGFR expression was noted in 73 patients (88.0%). There were no significant differences in mean ages, gender, tumor location, histological type, tumor depth, lymph node metastasis, retrieved lymph node number, vascular invasion, perineural invasion, pre-operative serum CEA level, and post-operative serum CEA level between patients with positive EGFR expression and those with negative EGFR expression. Moreover, OS (22.08 vs. 24.70 months, *P* = 0.523) and PFS (9.65 vs. 7.44 months, *P* = 0.417) were not significantly different between patients with positive EGFR expression and those negative EGFR expression. The differences of clinical and pathological data regarding the patients with metachronous mCRC and the patients with synchronous mCRC are summarized in Additional file 1: Table S1.

**Table 2:** **Baseline characteristics of synchronous metastatic colorectal cancer patients by EGFR expression and *KRAS *** **mutation status**

**Characteristic**	**EGFR positive (%)**	**EGFR negative (%)**	***P *** **value**	***KRAS *** **WT**^**a **^**(%)**	***KRAS *** **Mut**^**b **^**(%)**	***P *** **value**
	**N = 73 (88.0%)**	**N = 17 (12.0%)**		**N = 63 (58.9%)**	**N = 44 (4119%)**	
**Age (years, mean ± SD)**	61.14 ± 11.81	65.80 ± 8.05	0.231	61.83 ± 10.49	61.86 ± 13.93	0.987
**Gender**			0.787			0.119
Male	47 (64.4)	6 (60.0)		35 (55.6)	31 (70.5)	
Female	26 (35.6)	4 (40.0)		28 (44.4)	13 (29.5)	
**Tumor size**			0.047			0.134
≥5 cm	39 (53.4)	2 (20.0)		28 (44.4)	26 (59.1)	
<5 cm	34 (46.6)	8 (80.0)		35 (55.6)	18 (40.9)	
**Tumor location**			0.055			0.098
Colon	62 (84.9)	6 (60.0)		46 (73.0)	38 (86.4)	
Rectum	11 (15.1)	4 (40.0)		17 (27.0)	6 (13.6)	
**Histology**			0.862			*0.873*
Well	2 (2.7)	0 (00.0)		2 (3.2)	1 (2.3)	0.873
Moderately	58 (79.5)	8 (80.0)		49 (77.8)	36 (81.8)	
Poorly	13 (17.8)	2 (20.0)		12 (19.0)	7 (15.9)	
**Histology**			0.866			0.676
Well + Moderately	60 (82.2)	8 (80.0)		51 (81.0)	37 (84.1)	
Poorly	13 (17.8)	2 (20.0)		12 (19.0)	7 (15.9)	
**Tumor depth**			0.792			0.734
T1	1 (1.4)	0 (0.0)		1 (1.6)	0 (0.0)	
T2	3 (4.1)	1 (10.0)		3 (4.8)	3 (6.8)	
T3	48 (65.7)	7 (70.0)		45 (71.4)	29 (65.9)	
T4	21 (28.8)	2 (20.0)		14 (22.2)	12 (27.3)	
**Lymph nodes metastases**			0.407			0.824
N0	17 (23.2)	4 (40.0)		14 (22.2)	10 (22.7)	
N1	28 (38.4)	2 (20.0)		25 (39.7)	15 (34.1)	
N2	28 (38.4)	4 (40.0)		24 (38.1)	19 (43.2)	
**Retrived LN**^**c**^	17.09 ± 8.04	14.40 ± 5.85	0.313	16.31 ± 8.36	16.25 ± 8.17	0.971
**Vascular invasion**			0.756			0.863
Yes	33 (45.2)	4 (40.0)		29 (46.0)	21 (47.7)	
No	40 (54.8)	6 (60.0)		34 (54.0)	23 (52.3)	
**Perineurial invasion**			0.968			0.539
Yes	36 (49.3)	5 (50.0)		32 (50.8)	25 (56.8)	0.539
No	37 (50.7)	5 (50.0)		31 (49.2)	19 (43.2)	
**Pre-op serum CEA**^**b **^**level**			0.496			0.827
≥5 ng/ml	58 (79.5)	7 (70.0)		49 (77.8)	35 (79.5)	
<5 ng/ml	15 (20.5)	3 (30.0)		14 (22.2)	9 (20.5)	
**Post-op serum CEA**^**b **^**level**			0.568			0.799
≥5 ng/ml	21 (28.8)	4 (40.0)		43 (68.3)	29 (65.9)	
<5 ng/ml	46 (71.2)	6 (60.0)		20 (31.7)	15 (34.1)	
**Overall survival (months)**	22.08 ± 12.38	24.70 ± 9.91	0.523	23.04 ± 12.62	18.74 ± 11.39	0.074
**Progression-free survival (months)**	9.65 ± 7.44	11.66 ± 6.16	0.417	10.22 ± 7.14	7.95 ± 6.75	0.101

^a^WT: wild type; ^b^Mut: mutation; ^c^LN: lymph node.

*KRAS* mutation status was evaluated in 107 patients, and mutation was noted in 44 of those patients (41.1%). There were no significant differences in mean ages, gender, tumor location, histological type, tumor depth, lymph node metastasis, retrieved lymph node number, vascular invasion, perineural invasion, pre-operative serum CEA level, and post-operative serum CEA level between patients with wild-type *KRAS* and those with mutated *KRAS*. Moreover, OS (23.04 vs. 18.74 months, *P* = 0.074) and PFS (10.22 vs. 7.95 months, *P* = 0.101) were also not significantly different.

### Univariate and multivariable analyses of survival impact of EGFR expression and *KRAS* mutation in patients with metachronous mCRC

**Figure 2:**
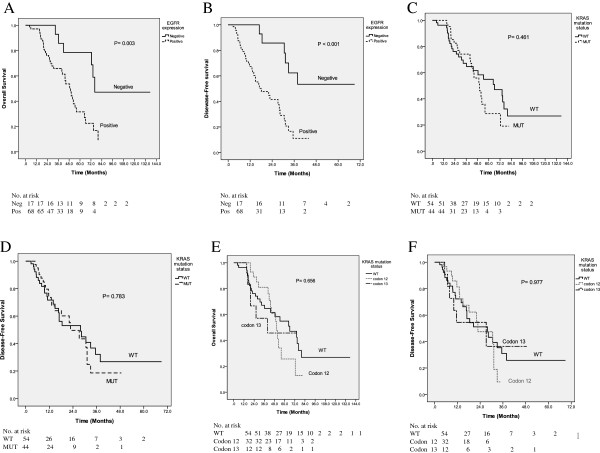
**The Kaplan-Meier survival curve for patients with metachronous mCRC. A**. Overall survival stratified by EGFR expression. **B**. Disease-free survival stratified by EGFR expression. **C**. Overall survival stratified by *KRAS* mutation status. **D**. Disease-free survival stratified by *KRAS* mutation status. **E**. Overall survival stratified by wild-type *KRAS*, codon 12, and codon 13. **F**. Disease-free survival stratified by wild-type *KRAS*, codon 12, and codon 13.

The univariate and multivariate analyses were performed to investigate independent prognostic factors for OS and DFS in patients with metachronous mCRC using the Cox proportional-hazards model (Table 3). Positive EGFR expression was demonstrated to be independent negative prognostic factors for OS (*P* = 0.028; HR, 3.090; 95% CI, 1.130–8.445) and DFS (*P* = 0.006; HR, 4.012; 95% CI, 1.477–10.900). However, *KRAS* mutation was not a significant prognostic factor for OS (*P* = 0.140; HR, 1.815; 95% CI, 0.823–4.004) and DFS (*P* = 0.260; HR, 1.440; 95% CI, 0.656–0.081). The Kaplan-Meier survival analysis also demonstrated that patients with positive EGFR expressions had worse OS (*P* = 0.003) and DFS (*P* < 0.001) (Figure 2A and 2B). The median OS times of patients with positive EGFR expression and those with negative EGFR expression were 49.50 and 76.20 months (*P* = 0.003; 95% CI, 41.223–57.777 and 52.175–99.920), respectively. The 5-year OS rates of patients with positive EGFR expression and those with negative EGFR expression were 23% and 79%, respectively. The median DFS times of patients with positive EGFR expression and those with negative EGFR expression were 20.96 and 50.17 months (*P* < 0.001; 95% CI, 17.216–24.708 and 38.822–61.510), respectively. The 3-year DFS rates of patients with positive EGFR expression and those with negative EGFR expression were 16% and 51%, respectively.

**Table 3:** Univariate and multivariable analysis of prognostic indicators on overall survival and disease-free survival for metachronous metastatic colorectal cancer patients (N = 98)

**Parameters**	**Overall survival**				**Disease-free survival**			
	**Univariate analysis**		**Multivariable analysis**		**Univariate analysis**		**Multivariable analysis**	
	**HR (95% CI)**	**P value**	**HR (95% CI)**	**P value**	**HR (95% CI)**	**P value**	**HR (95% CI)**	**P value**
**Age (years)**	0.691 (0.376–1.271)	0.235	0.678 (0.303–1.515)	0.343	0.829 (0.453–1.518)	0.544	0.990 (0.439–2.231)	0.980
(≥65 vs <65)								
**Sex**	0.933 (0.525–1.658)	0.933	0.848 (0.409–1.755)	0.656	1.018 (0.574–1.806)	0.951	0.912 (0.433–1.921)	0.808
(Male vs Female)								
**Location**	0.824 (0.454–1.495)	0.523	0.985 (0.449–2.164)	0.971	0.764 (0.421–1.388)	0.378	1.033 (0.477–2.237)	0.935
Rectum vs Colon								
**Tumor size**	0.897 (0.490–1.643)	0.725	1.088 (0.512–2.310)	0.826	0.896 (0.489–1.641)	0.722	1.043 (0.471–2.311)	0.917
(≥5 cm vs <5 cm)								
**Tumor depth**	0.972 (0.412–2.295)	0.949	0.699 (0.238–2.050)	0.514	1.047 (0.443–2.473)	0.917	1.030 (0.356–2.980)	0.956
T3 + T4 vs T1 + T2								
**LN metastasis**	1.062 (0.588–1.923)	0.844	0.989 (0.448–2.181)	0.570	1.090 (0.604–1.965)	0.776	0.813 (0.364–1.817)	0.614
Yes vs No								
**Histology**	605 (0.216–1.695)	0.339	0.628 (0.126–3.128)	0.766	0.625 (0.223–1.7448)	0.370	1.044 (0.210–5.200)	0.958
PD vs MD + WD								
**AJCC stage**	1.062 (0.586–1.923)	0.844			1.090 (0.604–1.965)	0.776		
III vs I&II								
**Vascular invasion**	1.643 (0.921–2.932)	0.093	1.160 (0.519–2.629)	0.707	1.555 (0.869–2.782)	0.137	1.297 (0.549–3.068)	0.553
Yes vs No								
**Perineurial invasion**	1.411 (0.785–2.536)	0.250	1.107 (0.470–2.611)	0.816	1.312 (0.731–2.354)	0.363	0.933 (0.369–2.361)	0.884
Yes vs No								
**Pre-op CEA (ng/ml)**	0.991 (0.530–1.851)	0.977	0.680 (0.289–1.603)	0.378	1.147 (0.616–2.134)	0.666	0.901 (0.377–2.154)	0.815
≥5/ vs <5								
**Post-op CEA (ng/ml)**	1.432 (0.763–2.688)	0.264	1.414 (0.561–3.562)	0.462	1,479 (0.788–2.779)	0.223	1.187 (0.454–3.101)	0.727
≥5 vs <5								
**EGFR expression**	3.577 (1.464–8.741)	0.005	3.090 (1.130–8.445)	0.028	4.609 (1.864–11.396)	0.001	4.012 (1.477–10.900)	0.006
Positive vs Negative								
**KRAS status**	1.249 (0.690–2.260)	0.462	1.815 (0.823–4.004)	0.140	1.066 (0.597–1.904)	0.829	1.440 (0.656–.081)	0.260
Mut vs WT								

The Kaplan-Meier survival analysis demonstrated no significant difference between patients with wild-type *KRAS* and those with mutated *KRAS* in terms of OS (*P* = 0.461) and DFS (*P* = 0.783) (Figure 2C and 2D). The median OS times of patients with wild-type *KRAS* and those with mutated *KRAS* were 66.10 and 50.30 months (*P* = 0.461; 95% CI, 39.430–92.770 and 40.770–59.830), respectively. The median DFS times of patients with wild-type *KRAS* and those with mutated *KRAS* were 37.90 and 22.80 months (*P* = 0.783; 95% CI, 11.120–44.680 and 14.470–31.130), respectively. Furthermore, we analyzed the OS and DFS of patients with wild-type *KRAS* (N = 54), mutated *KRAS* codon 12 (N = 32), and mutated *KRAS* codon 13 (N = 12). No significant difference was noted in terms of OS (*P* = 0.656) and DFS (*P* = 0.977) (Figures 2E and 2F).

### Univariate and multivariable analyses of survival impact of EGFR expression and *KRAS* mutation in patients with synchronous mCRC

**Figure 3:**
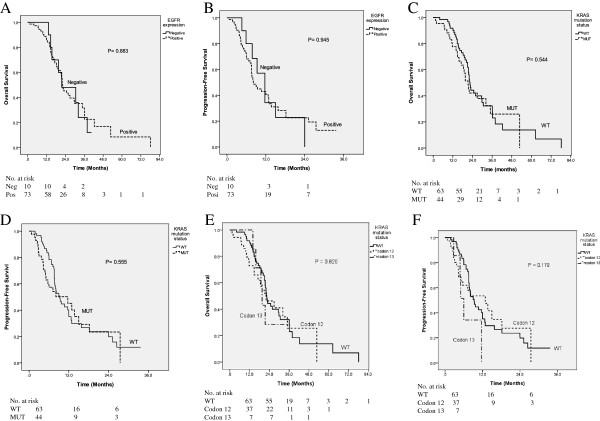
**The Kaplan-Meier survival curve for patients with synchronous mCRC. A**. Overall survival stratified by EGFR expression. **B**. Disease-free survival stratified by EGFR expression. **C**. Overall survival stratified by *KRAS* mutation status. **D**. Disease-free survival stratified by *KRAS* mutation status. **E**. Overall survival stratified by wild-type *KRAS*, codon 12, and codon 13. **F**. Disease-free survival stratified by wild-type *KRAS*, codon 12, and codon 13.

Univariate and multivariate analyses were performed to investigate the independent prognostic factors for OS and PFS in patients with synchronous mCRC using the Cox proportional-hazards model (Table 4). No variable was demonstrated to be an independent prognostic factor for OS and PFS in patients with synchronous mCRC. The Kaplan-Meier survival analysis demonstrated no significant difference between patients with positive EGFR expression and those with negative EGFR expression in terms of OS (*P* = 0.883) and PFS (*P* = 0.945) (Figure 3A and 3B). The median OS times of patients with positive EGFR expression and those with negative EGFR expression were 22.30 and 21.70 months (*P* = 0.883; 95% CI, 18.836–25.764 and 6.972–36.428), respectively. The median PFS times of patients with positive EGFR expression and those with negative EGFR expression were 8.20 and 11.70 months (*P* = 0.945; 95% CI, 6.356–10.044 and 8.425–14.975), respectively. In addition, the Kaplan-Meier survival analysis demonstrated no significant difference between patients with wild-type *KRAS* and those with mutated *KRAS* in terms of OS (*P* = 0.544) and PFS (*P* = 0.555) (Figure 3C and 3D). The median OS times of patients with wild-type *KRAS* and those with mutated *KRAS* were 22.50 and 21.30 months (*P* = 0.544; 95% CI, 21.036–23.964 and 17.967–24.633), respectively. The median PFS times of patients with wild-type *KRAS* and those with mutated *KRAS* were 9.30 and 11.70 months (*P* = 0.555; 95% CI, 7.395–11.205 and 4.696–18.704), respectively. Furthermore, we analyzed the OS and DFS of patients with wild-type *KRAS* (N = 63), mutated *KRAS* codon 12 (N = 37), and mutated *KRAS* codon 13 (N = 7). No significant difference was noted in terms of OS (P = 0.656) and PFS (P = 0.977) (Figure 3E and 3F).

**Table 4:** Univariate and multivariable analysis of prognostic indicators on overall survival and progress-free survival for synchronous metastatic colorectal cancer patients (N = 107)

**Parameters**	**Overall survival**				**Progression-free survival**			
	**Univariate analysis**		**Multivariable analysis**		**Univariate analysis**		**Multivariable analysis**	
	**HR (95% CI)**	**P value**	**HR (95% CI)**	**P value**	**HR (95% CI)**	**P value**	**HR**	**P value**
**Age (years)**	1.160 (0.697–1.929)	0.568	1.561 (0.812–2.999)	0.182	1.160 (0.697–1.929)	0.568	1.561 (0.812–2.999)	0.182
(≥65 vs <65)								
**Sex**	1.096 (0.661–1.818)	0.722	1.617 (0.790–3.307)	0.188	1.096 (0.661–1.818)	0.722	1.617 (0.790–3.307)	0.188
(Male vs Female)								
**Location**	0.668 (0.357–1.252)	0.209	0.540 (0.198–1.473)	0.229	0.668 (0.357–1.252)	0.209	0.540 (0.198–1.473)	0.229
Rectum vs Colon								
**Tumor size**	1.281 (0.784–2.094)	0.323	1.344 (0.683–2.644)	0.392	1.281 (0.784–2.094)	0.323	1.344 (0.683–2.644)	0.392
(≥5 cm vs <5 cm)								
**Tumor depth**	1.072 (0.333–3.456)	0.907	0.506 (0.107–2.396)	0.391	1.072 (0.333–3.456)	0.907	0.506 (0.107–2.396)	0.391
T3 + T4 vs T1 + T2								
**LN metastasis**	0.955 (0.541–1.684)	0.873	0.957 (0.488–1.879)	0.899	0.955 (0.541–1.684)	0.873	0.957 (0.488–1.879)	0.899
Yes vs No								
**Histology**	1.307 (0.678–2.520)	0.425	1.629 (0.655–4.052)	0.294	1.307 (0.678–2.520)	0.425	1.629 (0.655–4.052)	0.294
PD vs MD + WD								
**Vascular invasion**	1.092 (0.670–1.779)	0.724	0.826 (0.425–1.605)	0.573	1.092 (0.670–1.779)	0.724	0.826 (0.425–1.605)	0.573
Yes vs No								
**Perineurial invasion**	1.510 (0.918–2.485)	0.105	1.396 (0.740–2.636)	0.303	1.510 (0.918–2.485)	0.105	1.396 (0.740–2.636)	0.303
Yes vs No								
**Pre-op CEA (ng/ml)**	1.243 (0.623–2.482)	0.538	1.216 (0.418–3.540)	0.719	1.243 (0.623–2.482)	0.538	1.216 (0.418–3.540)	0.719
≥5/ vs <5								
**Post-op CEA (ng/ml)**	0.731 (0.425–1.257)	0.257	0.988 (0.412–2.370)	0.979	0.731 (0.425–1.257)	0.257	0.988 (0.412–2.370)	0.979
≥5 vs <5								
**EGFR expression**	0.945 (0.443–2.016)	0.883	0.648 (0.281–1.492)	0.308	0.945 (0.443–2.016)	0.883	0.648 (0.281–1.492)	0.308
Positive vs Negative								
**KRAS status**	1.167 (0.707–1.925)	0.546	1.051 (0.554–1.992)	0.879	1.167 (0.707–1.925)	0.546	1.051 (0.554–1.992)	0.879
Mut vs WT								

## Discussion

Of the 205 patients analyzed in this study, 98 patients had metachronous and 107 had synchronous mCRC. Positive EGFR expression was found in 80.5% patients through immunohistochemical analyses. The positive rate of EGFR expression in CRC was reported to be 25% to 82% [7]. *KRAS* mutation status was evaluated in 205 patients and mutation was noted in 88 of those patients (42.9%), in concordance with the mutation rate of *KRAS* in CRC (35% to 42%) [19]. In our patients with metachronous mCRC, EGFR expression was associated with differentiation grade of the tumor, with more moderate differentiation in patients with positive EGFR expression (*P* = 0.028), in accordance with the report of Andreyev et al. [25]. However, the association was not noted in our synchronous mCRC patients. The association between histological grade and EGFR expression is still controversial [6-8,33,34].

For the prognostic value of EGFR for patients with metachronous mCRC, we have demonstrated EGFR as an independent negative prognostic factor for OS and DFS by multivariate Cox proportional-hazards model. The Kaplan-Meier survival analysis also showed that patients with positive EGFR expression had worse OS and DFS. Galizia et al. [7] have shown that there is strong association between disease-specific survival and EGFR expression status, and a more than 10-fold risk of cancer related death in patients with positive EGFR expression compared with patients with negative EGFR expression. The difference was even stronger in patients with Duke’s C and D colon cancer than in those with Duke’s A and B colon cancer [7]. Ljuslinder et al. [6] have shown an association between worse outcomes and higher EGFR expression at invasive margin. Giralt et al. [9] evaluated the relationship between prognosis and EGFR expression in patients with locally advanced rectal cancer (LARC) receiving preoperative radiotherapy, and they found that the pathological response rate was lower in patients with positive EGFR expression than in those with negative EGFR expression. Azria et al. [10] conducted a similar study to evaluate the prognostic impact of EGFR expression on locoregional recurrence in patients with LARC receiving preoperative radiotherapy. The locoregional recurrence rate was higher in patients with EFGR extent ≧25% than in patients with EFGR ≦25% (20% vs. 7%). The locoregional recurrence-free survival rate at 2 years was 94% and 84%, respectively (*P* = 0.06). EFGR extent ≧25% was a significant factor for locoregional recurrence (*P* = 0.037; HR, 7.18; 95% CI, 1.17–46). Theodoropoulos et al. [34] reported a significant association between high EGFR expression and advanced T3 and T4 stages (*P* = 0.001), which implied that EGFR overexpression was associated with tumor invasion. Furthermore, they also demonstrated a trend between positive EGFR expression and poorer OS. Deng et al. [35] reported a significant association between high EGFR expression in primary tumor and poorer OS (*P* = 0.046); however, the association was not noted in stage IV patients, which is in agreement with our present study. The association between EFGR expression and worse survival has also been noted in other malignancies, such as gastric cancer [36,37], esophageal cancer [38], and breast cancer [39]. In contrast, Spano et al. [8] and McKay et al. [33] reported no significant association between EGFR expression and survival.

Through the multivariate Cox proportional-hazards analyses and Kaplan-Meier survival analysis used in this study, *KRAS* mutation status was found not to be a significant independent prognostic factor of OS, DFS, and PFS for patients with metachronous mCRC and synchronous mCRC. Roth et al. [19] reported a mutated rate of 37% of *KRAS* mutation from 1299 patients with stages II and III colon cancer. No significant association between survival (OS and relapse-free survival) and *KRAS* mutation status was demonstrated. Moreover, no difference was noted between survival (OS and relapse-free survival) and type of *KRAS* mutation stratified by condon 12 and 13 in patients with stages II and III colon cancer, which is in agreement with our analyses of patients with metachronous mCRC. Rose et al. [20] assessed the survival impact of *KRAS* mutation status in 110 patients with metachronous and synchronous mCRC. The OS of patients with metachronous and those with synchronous mCRC was not influenced by *KRAS* mutation status (*P* = 0.55 and 0.37, respectively), which is also consistent with our present study. Three studies [21-23] from Asia evaluating the survival impact of *KRAS* mutation status in CRC patients also showed that there was no prognostic value of *KRAS* mutation status for OS and PFS. Recently, a systemic review and meta-analysis regarding the prognostic value of *KRAS* mutation status demonstrated that there was no association between *KRAS* mutation status and the prognosis of patients with CRC [24]. In contrast, 2 large collaborative Kirsten Ras in Colorectal Cancer Collaborative Group (RASCAL) studies [25,26] evaluated the prognostic role in CRC. It was concluded in these studies that mutated *KRAS* were significantly associated with an increased risk of relapse and death and a significant association between failure-free survival and G12V of mutated *KRAS* in Duke’s C patients. Richman et al. [27] reported similar PFS and worse OS in patients with mutated *KRAS* compared to patients with wild-type *KRAS*. Roth et al. indicated that the *KRAS* mutations were assessed by each referring center according to three types of methodologies, and meta-analysis results could be affected by variations between trials [19]. In a meta-analysis assessing the predictive and prognostic value of *KRAS* mutations in CRC patients treated with cetuximab, it was concluded that mCRC patients with mutated *KRAS* could have worse PFS and OS when treated with cetuximab [28].

To our best knowledge, this study is the first to evaluate the prognostic values of EGFR expression and *KRAS* mutation simultaneously in patients with metachronous and synchronous mCRC. However, there are some limitations to the present study. First, the present study is a single-institution retrospective study. The study’s relatively small sample size is another limitation. Third, the *KRAS* mutation status was evaluated only as wild-type and mutant (codon 12 and codon 13) and we did not analyze other rare mutation types.

## Conclusion

In conclusion, we have demonstrated that EGFR expression has prognostic value only for patients with metachronous mCRC, while having no such value for patients with synchronous mCRC. Our data indicate EGFR as an independent negative prognostic factor for OS and DFS in patients with metachronous mCRC. Analyzing EGFR expression may help identify high-risk patients requiring more aggressive therapeutic modalities in the setting of metachronous mCRC. However, *KRAS* mutation did not have prognostic value for patients with metachronous or synchronous mCRC.

## Abbreviations

EGFR: Epidermal growth factor receptor; CRC: Colorectal cancer; DFS: Disease-free survival; OS: Overall survival; PFS: Progression-free survival; CEA: Carcinoembryonic antigen; AJCC: American joint commission on cancer; IHC: Immunohistochemistry; PD: Poorly differentiated; MD: Moderately differentiated; WD: Well differentiated.

## Competing interests

The authors declare that they have no competing interest.

## Authors’ contributions

CWH analyzed the data and wrote the manuscript. HLT, CMH, CJM, CYL, CHK and DCW made substantial contributions in data acquisition, statistical analyses, and data interpretation, and helped in manuscript preparation. YTC and CYC participated in making formalin-fixed and paraffin-embedded tissue blocks, immunohistochemical staining of EGFR, and interpretation of EGFR expression. JYW participated in study design and coordination. All authors have read and approved the final manuscript.

## Pre-publication history

The pre-publication history for this paper can be accessed here:

http://www.biomedcentral.com/1471-2407/13/599/prepub

## Supplementary Material

Additional file 1: Table S1 Baseline characteristics of metachronous and synchronous metastatic colorectal cancer patients. Click here for file
