# Supplementary material for: The prognostic values of EGFR expression and KRAS mutation in patients with synchronous or metachronous metastatic colorectal cancer
Source: BMC Cancer. 2013 Dec 13;13:599. doi: 10.1186/1471-2407-13-599 (PMC3878756; doi:10.1186/1471-2407-13-599)
Supplement: Additional file 1: Table S1 — Baseline characteristics of metachronous and synchronous metastatic colorectal cancer patients. [file 1471-2407-13-599-S1.docx]

**Additional file 1: Table S1. Baseline characteristics of metachronous and synchronous metastatic colorectal cancer patients**

| **Characteristic** | **Metachronous (%)**  N = 98 (47.8%) | **Synchronous (%)**  N = 107 (52.2%) | ***P* value** |
| --- | --- | --- | --- |
| **Age** (years, mean ± SD) | 59.62 ± 11.90 | 61.84 ± 11.96 | 0.185 |
| **Gender**  Male Female | 54 (55.1)  44 (44.9) | 66 (61.7)  41 (38.3) | 0.339 |
| **Tumor size** ≥5 cm <5 cm | 34 (34.7)  64 (65.3) | 53 (50.5)  52 (49.5) | 0.023 |
| **Tumor location** Colon Rectum | 61 (62.2)  37 (37.8) | 84 (78.5)  23 (21.5) | 0.011 |
| **Histology**  Well Moderately Poorly | 2 (2.0)  84 (85.8)  4 (12.2) | 3 (2.8)  85 (79.4)  19 (17.8) | 0.498 |
| **Histology**  Well+Moderately Poorly | 86 (87.8)  12 (12.2) | 88 (82.2)  19 (17.8)) | 0.271 |
| **Tumor depth** T1 T2 T3 T4 | 1 (1.0)  10 (10.2)  73 (74.5)  14 (14.3) | 1 (0.9)  6 (5.6)  74 (69.2)  26 (24.3) | 0.239 |
| **Lymph Nodes metastases** N0 N1 N2 | 34 (34.7)  43 (43.9)  21 (21.4) | 24 (22.4)  40 (37.4)  43 (40.2) | 0.011 |
| **Retrived LN** | 15.33±9.57 | 16.28±8.24 | 0.481 |
| **Vascular invasion**  Yes  No  **Perineurial invasion** Yes No | 37 (37.8)  61 (62.2)  36 (36.7)  62 (63.3) | 50 (46.7)  57 (53.3)  57 (53.3)  50 (46.7) | 0.194  0.018 |
| **EFGR expression**  Positive  Negative | 67 (79.8)  17 (20.2) | 73(88.0)  10(12.0) | 0.151 |
| ***KRAS* status**  WT  Mut | 54(55.1)  44(44.9) | 63(58.9)  44(41.1) | 0.585 |
| **Overall survival (months)** | 40.24±23.85 | 21.27±12.26 | <0.001 |
